# Dysfunctional autophagy in RPE, a contributing factor in age-related macular degeneration

**DOI:** 10.1038/cddis.2016.453

**Published:** 2017-01-05

**Authors:** Nady Golestaneh, Yi Chu, Yang-Yu Xiao, Gianna L Stoleru, Alexander C Theos

**Affiliations:** 1 Department of Ophthalmology, Georgetown University Medical Center, Washington, DC, USA; 2 Department of Neurology, Georgetown University Medical Center, Washington, DC, USA; 3 Department of Biochemistry and Molecular & Cellular Biology, Georgetown University Medical Center, Washington, DC, USA; 4 Department of Human Science, Georgetown University, Washington, DC, USA

## Abstract

Age-related macular degeneration (AMD) is a devastating neurodegenerative disease and a major cause of blindness in the developed world. Owing to its complexity and the lack of an adequate human model that recapitulates key aspects of the disease, the molecular mechanisms of AMD pathogenesis remain poorly understood. Here we show that cultured human retinal pigment epithelium (RPE) from AMD donors (AMD RPE) are functionally impaired and exhibit distinct phenotypes compared with RPE cultured from normal donors (normal RPE). Accumulation of lipid droplets and glycogen granules, disintegration of mitochondria, and an increase in autophagosomes were observed in AMD RPE cultures. Compared with normal RPE, AMD RPE exhibit increased susceptibility to oxidative stress, produce higher levels of reactive oxygen species (ROS) under stress conditions, and showed reduced mitochondrial activity. Measurement of the ratio of LC3-II/ LC3-I, revealed impaired autophagy in AMD RPE as compared with normal RPE. Autophagic flux was also reduced in AMD RPE as compared with normal RPE, as shown by inability of AMD RPE to downregulate p62 levels during starvation. Impaired autophagic pathways were further shown by analyzing late autophagic vesicles; immunostaining with lysosome-associated membrane protein 1 (LAMP-1) antibody revealed enlarged and annular LAMP-1-positive organelles in AMD RPE as opposed to smaller discrete puncta observed in normal RPE. Our study provides insights into AMD cellular and molecular mechanisms, proposes dysfunctional autophagy as an underlying mechanism contributing to the pathophysiology of the disease, and opens up new avenues for development of novel treatment strategies.

Age-related macular degeneration (AMD) is a major cause of vision loss among elderly in developed countries worldwide. AMD affects approximately 30–50 million people, ^1, 2^ and its prevalence is expected to double by 2050.^3^

AMD initially affects the retinal pigment epithelium (RPE), a monolayer of pigmented and polarized central nervous system (CNS) tissue, and over time, leads to secondary loss of photoreceptor cells.^2, 4^

AMD is a multifactorial disease^3^ and its pathogenesis remains largely elusive. Mounting evidence suggests a complex interaction of genetic, environmental and metabolic factors contributing to the pathology of AMD.^5^ Impaired RPE function in dry AMD causes the formation of extracellular deposits called drusen,^6^ which accumulate between the RPE and Bruch's membrane (BM). The wet form of AMD involves choroidal neovascularization followed by formation of a disciform scar.^7^ Dry AMD could transform to the wet form at the latest stage of the disease.^2^ However, a patient could also present both forms at different stages of AMD.

The RPE has important roles in retinal homeostasis. It functions as a retinal blood barrier, a source of polarized growth factor release and transporter of ions, water and metabolic products from the subretinal space to the blood ^8, 9^ The RPE delivers blood-derived nutrients to photoreceptors, absorbs light and performs phagocytosis of the outer segments of photoreceptors.^10^

Numerous studies have reported on age-related physiological changes in RPE,^11^ including mitochondrial DNA damage,^12^ accumulation of lipofuscin,^13^ elevated amyloid *β* production,^14^ enhanced tissue factor (TF) expression,^15^ increased acidic beta-galactosidase activity suggestive of lysosomal dysfunction^16^ and altered expression of RPE structural proteins.^17^

Autophagy is a lysosome-mediated degradation process for non-essential or damaged cellular constituents to supply the cell with energy and to maintain homeostasis.^18, 19^ Recently, much interest has focused on the role of autophagy in health and disease.^20, 21^ Moreover, lipid droplets have been identified as an autophagy substrate^22^ and the impact of autophagy on mitochondrial function has been discussed.^23, 24^ The latter is particularly relevant to degenerative diseases, such as AMD, in which oxidative stress occurs over time. Recently, dysregulated autophagy in RPE was shown to increase susceptibility to oxidative stress and AMD.^25^ Another study reported that activation of autophagy clears ELAVL1/HuR-mediated accumulation of SQSTM1/p62 during proteasomal inhibition in human RPE cells.^26^

Several reviews also propose a role for autophagy in AMD,^27, 28, 29^ however, direct evidence for altered autophagic function in the pathophysiology of AMD has yet to be documented in an *in vitro* cellular model.

Here, we analyzed the phenotype and function of RPE cultures established from human donor eyes. In this novel system for studying AMD, we show that autophagy is selectively dysregulated in AMD RPE contributing to disease phenotypes.

## Results

### Human RPE from AMD donors exhibit distinct disease phenotypes

We developed AMD RPE cultures by isolating RPE from macula region of five AMD donors (AMD RPE) and five normal donors (normal RPE) according to established protocols.^30^ We purified RPE cells by magnetic cell sorting, using specific antibodies as described below and cultured them at 5% oxygen concentration to avoid oxidative stress. We performed the genetic study of single-nucleotide polymorphisms (SNPs) for AMD susceptibility loci (Table 1). Interestingly, AMD donor# 9, which had a protective allele, nevertheless developed AMD, possibly due to heavy smoking (40 years of two packs of cigarettes per day), whereas control donors #6 and #10 despite presenting the AMD susceptibility loci did not develop disease, providing further evidence for the multifactorial origin of AMD.

**Figure 1:**
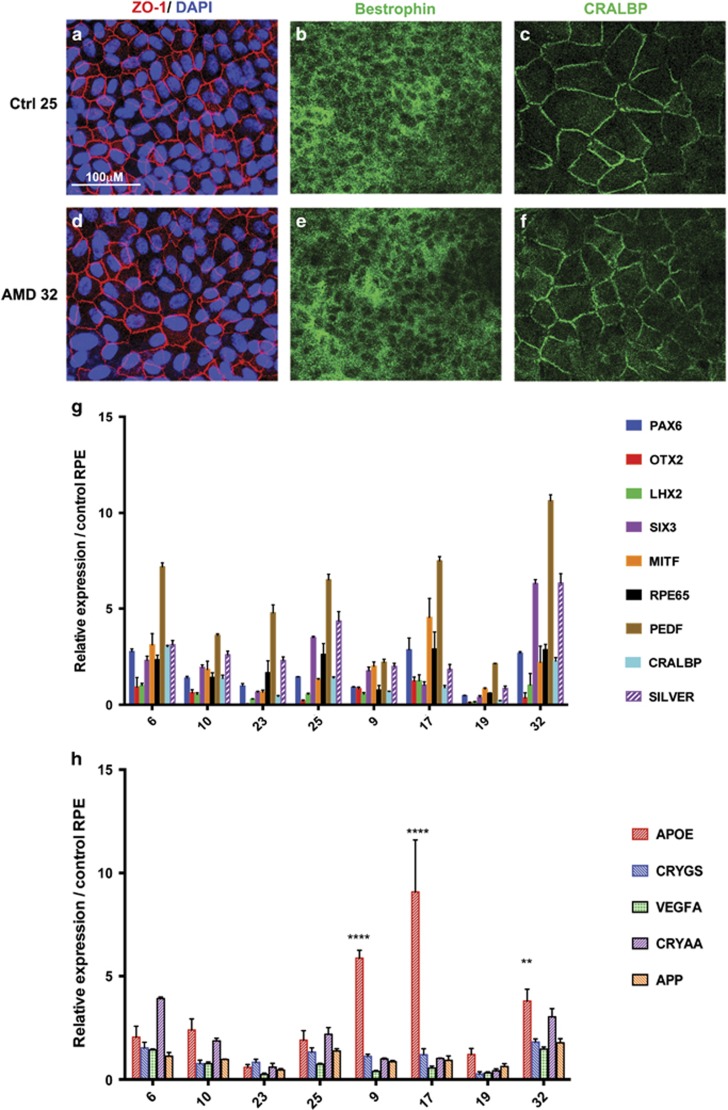
(**a**-**f**) Isolation and characterization of RPE from donors. The isolated RPE from AMD and control donors express RPE-specific proteins ZO-1, Bestrophin and CRALBP. A representative image of immunostaining is shown for each group. Bar represents 100 *μ*M. (**g**) Gene expression analysis by qRT-PCR confirming the expression of RPE marker genes in the RPE isolated from donors (controls 6, 10, 23 and 25; AMD 9, 17, 19 and 32). (**h**) Gene expression analysis of AMD-associated genes in AMD and normal RPE. **P<0.01, ****P<0.0001

SNPs analysis was performed to obtain genetic information for AMD-associated loci for our samples, and not to make any association of genetics with cellular phenotypes because of the small sample size in our study. Table 1 summarizes the age, gender and genetic characteristics of the cultured RPE. We also analyzed the cells for expression of specific RPE proteins and genes (Figure 1). Our stained RPE were positive for ZO-1, Bestrophin and the retinaldehyde-binding protein (CRALB), which binds to 11-cis-retinal in the visual cycle, and whose function is associated with normal dark adaptation.^31^

Gene expression analysis was performed by qPCR and showed expression of all RPE-specific genes in both the AMD and normal RPE cultures. We also analyzed the expression of AMD-associated genes and interestingly found that the expression of apolipoprotein E (APOE) was increased in three out of four tested AMD RPE as compared with four normal RPE. APOE is a prominent component of drusen, has a central role in lipid metabolism and is believed to be involved in lipid trafficking in AMD.^32^

**Figure 2:**
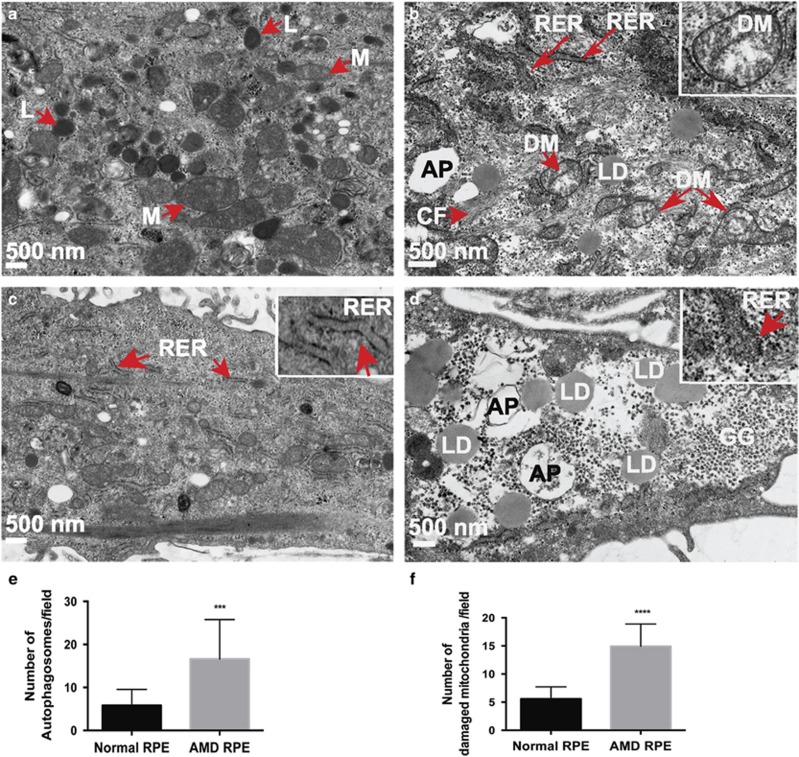
AMD RPE exhibit disease phenotypes. (**a-d**) EM images of normal 10 (**a** and **c**) and AMD 9 (**b** and **d**) RPE showing disease phenotypes. Red arrows indicate the observed morphological differences. Higher magnification insets show the observed phenotypes (in **b** and **d**), in comparison with the normal cellular structures (in **c**). L, lysosomes; M, mitochondria; RER, rough ER; LD, lipid droplets; F, cytoskeletal fascicles. (**e** and **f**) Number of APs and damaged mitochondria were counted (*n*=3) in four random regions per cell. The images used for counting were all the same size and same magnification. The mean±S.D. and the *P*-values were calculated for statistical significance. *P*-value equals 0.0002 (***) and 0.0001 (****) for (**e** and **f**), respectively

Electron microscopy (EM) imaging (Figure 2) revealed that AMD RPE cultures exhibited distinct disease phenotypes compared with normal RPE. AMD RPE (Figures 2b and d) contained less dense cytoplasm, a higher content of lipid droplets, increased number of glycogen granules and enlarged autophagosomes (APs). The space between the two membranes of the rough endoplasmic reticulum (RER) appeared to be wider and irregular in AMD RPE (Figure 2b and inset 2d), whereas it appeared rather uniform in normal RPE (Figure 2c and inset 2c). Cytoskeletal filaments formed fascicles and appeared condensed (Figure 2b) and mitochondria appeared disintegrated in AMD RPE compared with normal RPE (Figure 2b). We also counted the number of APs and damaged mitochondria and performed a blind comparison of the EM images of normal and AMD RPE (*n*=3). For each sample, we counted four random regions per cell. The images used for counting were all the same size and same magnification. The mean±S.D. and the *P*-values were calculated for statistical significance. We observed significant difference between the numbers of APs and disintegrated mitochondria (DM) in AMD RPE as compared with normal RPE (Figures 2e and f).

**Figure 3:**
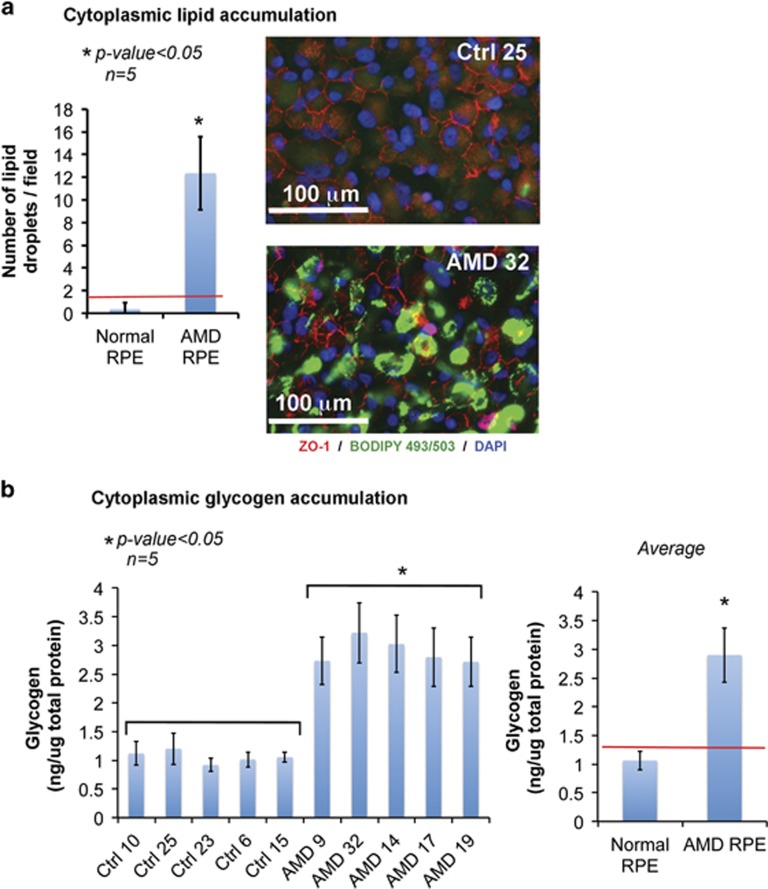
Lipid and glycogen accumulation in AMD RPE. (**a**) (Left): graph showing the quantification of lipid droplets in normal and AMD RPE, as determined by counting the lipid droplets in three random fields of stained images (shown in right panel), in the five normal and five AMD RPE (*n*=5). Asterisk (*) indicates statistically significant difference in number of lipid droplets between control and AMD RPE. (**a**) (Right): analysis of cytoplasmic lipid by fluorescence staining in control and AMD RPE, showing higher intensity of lipid staining in the AMD RPE. (**b**) (Left): measurement of glycogen accumulation by colorimetric assay showing statistically significant higher concentration in the AMD RPE compared with control RPE (*n*=5). (**b**) (Right): graph of the average glycogen concentrations calculated from five normal and five AMD RPE shown in the left graph. Red line indicates the threshold that separates AMD and normal RPE. Asterisk (*) indicates statistically significant differences in glycogen concentrations between the AMD and control RPE. *P*-values in (**a** and **b**) are determined by ANOVA followed by Tukey's test, with *P*<0.05

Quantification of lipid droplets and glycogen granules by fluorescence staining and colorimetric assay, respectively, revealed higher levels of lipid droplets and glycogen granules in AMD RPE as compared with normal RPE, further confirming the EM phenotypes (Figures 3a and b). The distinct phenotypes identified in AMD RPE support the use of these cells as an *in vitro* model to study the underlying mechanisms responsible for the observed phenotypes.

### AMD RPE show increased susceptibility to oxidative stress, produce higher levels of reactive oxygen species (ROS) and exhibit lower mitochondrial activity

To assess the functional consequences of the observed phenotypes, we first established chronic oxidative stress conditions using a series of increasing H_2_O_2_ concentrations. Under these conditions, RPE cells can be cultured up to 48 h with increasing doses of H_2_O_2_ to study their susceptibility to oxidative stress.

**Figure 4:**
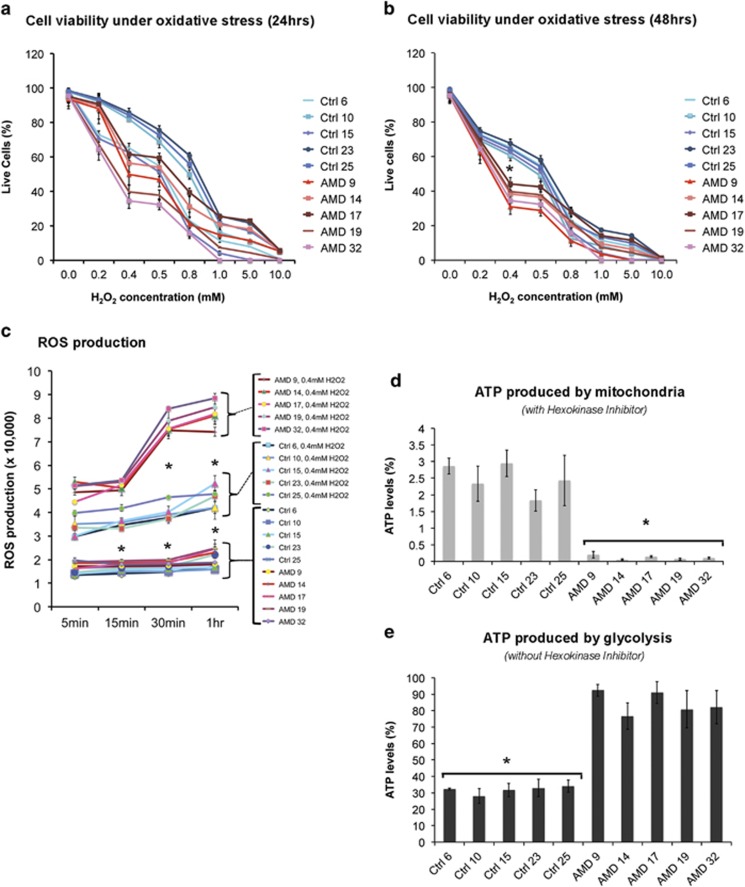
AMD RPE are more susceptible to oxidative stress and show lower mitochondrial activity. (**a** and **b**) Cell viability assays of AMD and control RPE treated with increasing concentrations of H_2_O_2_ for 24 h (**a**) and 48 h (**b**). Higher susceptibility of the AMD RPE under oxidative stress conditions is observed in 48 h. (**c**) ROS production under stress is significantly higher in AMD RPE. (**d** and **e**) AMD RPE have significantly lower mitochondrial activity, as indicated by their ATP levels measured by a luminescence assay in the presence of hexokinase inhibitor. (**d**) ATP levels produced by mitochondria are significantly lower in AMD RPE as measured in the presence of hexokinase inhibitor. (**e**) ATP levels produced by glycolysis are higher in AMD RPE as measured in the absence of hexokinase inhibitor. The difference in ATP levels measured in the presence (**d**) and absence (**e**) of hexokinase inhibitor show glycolysis as the major source of ATP production in AMD RPE. Asterisks (*) indicate statistical significance, determined by the ANOVA analysis followed by Tukey's test (*P*-value<0.05, *n*=5 for each sample)

Cell viability assays using 0.2–10 mM of H_2_O_2_ for 24 and 48 h revealed that AMD RPE and normal RPE are affected differently by oxidative stress. Our data demonstrated that while H_2_O_2_ treatment for 24 h only partially separated the AMD RPE from normal RPE (Figure 4a), 48 h of H_2_O_2_ treatment revealed significantly higher vulnerability for AMD RPE (*P*-value: 0.01). The AMD RPE died faster and at a lower concentration of H_2_O_2_ compared with normal RPE (Figure 4b).

It has been proposed that mitochondrial dysfunction leads to elevated ROS production.^33^ To examine whether structural defects observed in mitochondria (Figure 2) correlate with elevated oxidative stress, we measured ROS production in the presence of 0.4 mM H_2_O_2_ for 5 min to 1 h in AMD RPE and normal RPE. Our data showed that AMD RPE produce significantly higher ROS levels than normal RPE under oxidative stress (Figure 4c).

A recent report showed that mtDNA damage positively correlated with the severity of AMD, whereas repair capacity was negatively correlated.^12^ To analyze mitochondrial activity in AMD and normal RPE, we measured ATP levels. When we treated the cells with hexokinase inhibitor to block the glycolytic ATP production, the ATP produced by mitochondria was significantly lower in AMD RPE (Figure 4d). Conversely, ATP levels measured in the absence of hexokinase inhibitor revealed that glycolytic ATP production is significantly higher in AMD compared with normal RPE (Figure 4e), suggesting that in AMD RPE, ATP is produced primarily through glycolysis. These deficits in mitochondrial function correlate well with the altered mitochondrial morphology observed by EM (Figure 2b).

### Autophagy is dysfunctional in AMD RPE

**Figure 5:**
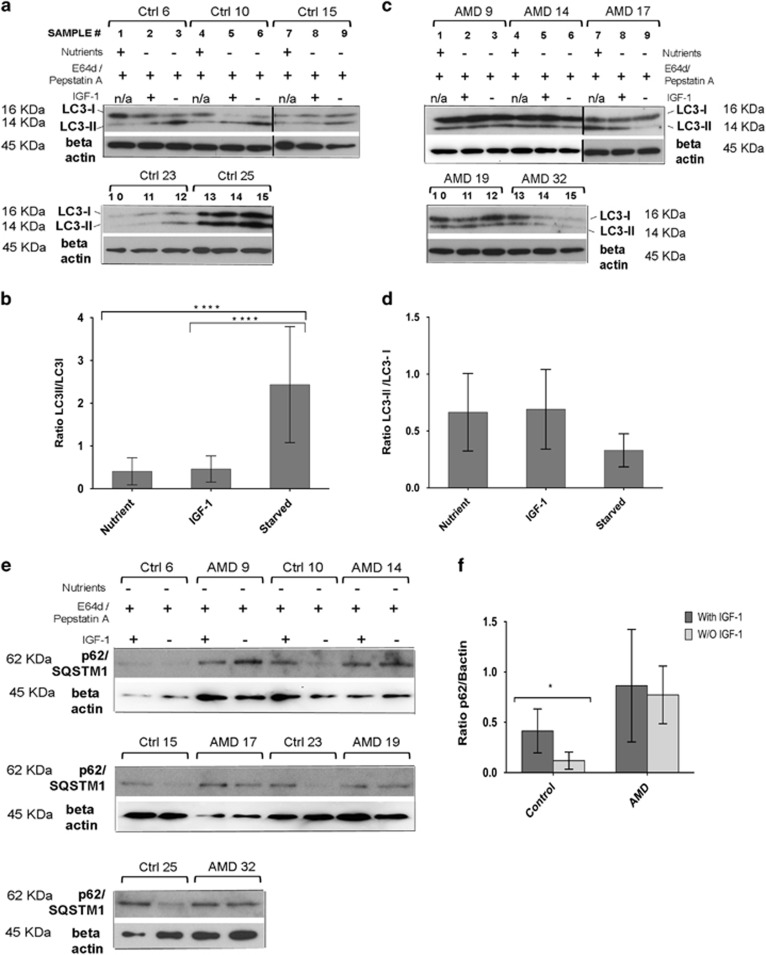
Autophagy is dysfunctional in AMD RPE. (**a** and **c**) Analysis of autophagy dynamics in normal RPE (**a**), and AMD RPE (**c**), *n*=5. LC3 immunoblots of control and AMD RPE under three different conditions, nutrient, starvation in the presence of IGF-1, and starvation in the absence of IGF-1 are shown. Beta actin is used as a normalization control. Spliced membranes are indicated by the vertical lines. (**b** and **d**) The ratios of the LC3-II/LC3-I levels as determined by densitometry are illustrated in the graphs, showing that an increase in autophagy dynamics in the absence of IGF-1 under starvation conditions is observed only in normal, but not in AMD RPE. Densitometry was performed on three repeats of the experiment for each sample in five normal and five AMD RPE (**b** and **d**). Asterisks in (**b**) represent *P*-value <0.0001 of LC3-II/LC3-I ratios as determined by one-way Anova followed by Tukey's test. (**e** and **f**) Autophagic flux is lower in AMD RPE as compared with normal RPE. (**e**) Immunoblot of p62, demonstrating lower autophagic flux in AMD RPE as shown by inability of AMD RPE to downregulate p62 levels during starvation in the absence of IGF-1. Beta actin is used as a normalization control. (**f**) Relative expression of p62 in control and AMD RPE in the presence and absence of IGF-1, as determined by densitometry analysis of the immunoblot in (**e**), *n*=5. The asterisks (*) indicate statistical significance determined by ANOVA followed by Tukey's test (*P*<0.05)

To investigate the biological relevance of AP accumulation observed in our EM data (Figures 2b and d) and to directly study the role of autophagy in the pathophysiology of AMD, we measured autophagy by inducing nutrient starvation in the presence of lysosomal inhibitors and in the presence or absence of the insulin growth factor (IGF-1). A commonly accepted method to monitor autophagic flux is to assay processing of ubiquitin-like microtubule-associated protein 1 light chain LC3-I, which after lipidation becomes LC3-II, is inserted into the inner and outer membranes of the AP and is eventually degraded.^34^ Therefore, to obtain an accurate measurement of the endogenous levels of LC3-II, we pre-treated the AMD and normal RPE with E64d and pepstatin A^35^ to inhibit lysosomal degradation. Under these conditions, the levels of the lipidated form of LC3-II relative to unprocessed LC3-I are an accurate measurement of autophagic flux. Our results revealed that normal RPE rapidly induced autophagy after starvation in the absence of IGF-1 (Figures 5a and b), whereas AMD RPE failed to increase the ratio of LC3-II/LC3-I, under the same conditions (Figures 5c and d). Therefore, the addition of IGF-1, expected to suppress autophagy through activation of the AKT/mTOR signaling in normal cells, did not seem to decrease the ratio of LC3-II/LC3-I in AMD RPE (Figures 5c and d), as shown by the densitometry of western blots of three independent experiments (Figures 5b–d). These observations suggest that induction of autophagy in response to starvation is impaired or limited in AMD RPE. Although autophagy dynamics could vary from sample to sample and based on the severity of the disease phenotypes and stage of the disease in each sample, we observed a general trend of lower autophagy dynamics in AMD as compared with normal RPE.

p62, also called sequestosome 1 (SQSTM1), possesses a short LC3 interaction region that facilitates direct interaction with LC3 and causes p62 degradation by autophagy.^36, 37, 38^ p62 is known to accumulate in cytoplasmic and nuclear ubiquitinated protein aggregates in various neurodegenerative diseases.^39^ As p62 degradation depends on autophagy, the level of p62 protein is another indicator of autophagic flux.^40^

To investigate autophagic flux in AMD RPE, we compared p62 levels in AMD RPE and normal RPE under the same experimental conditions as described above. As shown in Figures 5e and f, under starvation and in the absence of IGF-1 the levels of p62 were decreased in normal RPE as compared with p62 levels under starvation and in the presence of IGF-1. However, under the same conditions, AMD RPE were unable to downregulate p62 levels (Figure 5f, *n*=5).

Therefore, despite the observed accumulation of APs by EM (Figures 2b and d), AMD RPE cultures failed to induce autophagy during starvation suggesting dysfunctional autophagy and significantly lower autophagic flux in AMD RPE.

**Figure 6:**
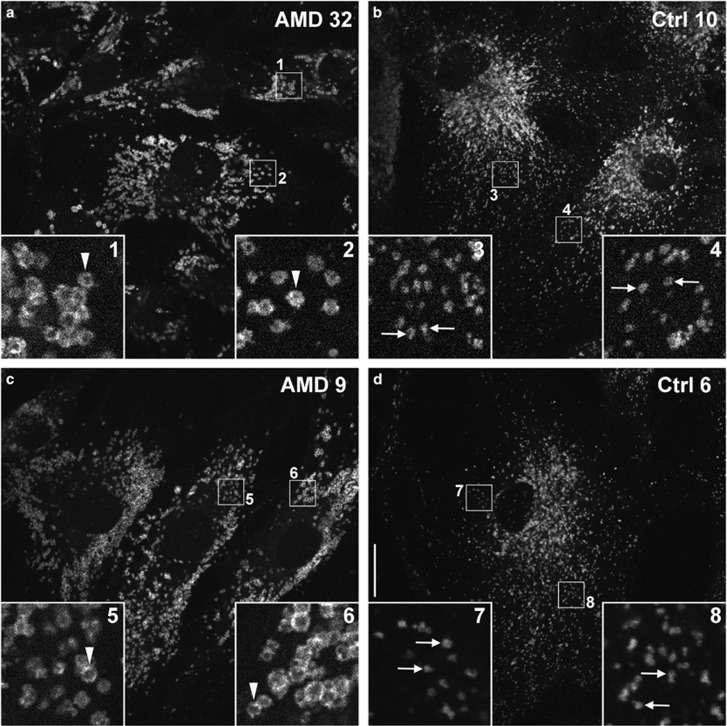
Autophagolysosomes are swollen in AMD RPE. Swollen LAMP-1-positive organelles, commonly indicative of defective lysosomal function, are consistently observed in AMD RPE (white arrowheads, AMD 32 (**a**) and AMD 9 (**c**)), but not in healthy RPE (white arrows, Ctrl 10 (**b**) and Ctrl 6 (**d**)). Insets (1–8) are × 5 magnifications of indicated boxed regions, scale bar represents 20 *μ*m

We further analyzed the late autophagic vesicles, autolysosomes by immunostaining with lysosomal-associated membrane protein 1 (LAMP-1) antibody in AMD and normal RPE cultures. Our data showed that LAMP-1-positive organelles in AMD RPE are enlarged and annular, as opposed to the smaller discrete puncta observed in normal RPE (compare arrowed structures with arrowheads in magnified insets within Figure 6). This enlarged morphology suggests inefficient degradation of cellular debris or inefficient recycling of membranes within the lysosomes of AMD RPE. Together, these observations strongly suggest dysfunctional autophagy in AMD RPE that translates into lower autophagic flux and accumulation of cytoplasmic debris.

## Discussion

Our study identifies morphological and functional differences between RPE cell cultures established from healthy human donors and donors with AMD. Hallmark morphological changes associated with AMD RPE included accumulation of APs, glycogen granules and lipid droplets, as well as DM. Functional analysis revealed increased vulnerability to oxidative stress, higher ROS production under oxidative stress and lower mitochondrial activity in AMD compared with normal RPE. Analysis of autophagic efficiency showed dysfunctional autophagic activity and inability to induce autophagy in response to starvation in AMD RPE. Collectively, our study suggests that impaired autophagy dynamics in RPE contributes to the pathophysiology of AMD. These observations provide insights into biochemical pathways that are affected in AMD and may have important implications for development of potential therapeutic interventions for this devastating neurodegenerative disease.

### Distinct AMD disease phenotypes

Despite the high prevalence of AMD, to date, there exists no disease altering treatment for dry AMD and millions of people worldwide continue to suffer from this debilitating disease.^1, 41^

A major limitation in understanding the pathophysiology of AMD is its complexity and the lack of a single animal model^42^ and absence of an *in vitro* model that could fully replicate key features of the human disease. This is primarily due to the multifactorial origin of AMD, such as human genetic polymorphisms and long-term exposure to environmental factors^43^ that induce epigenetic changes.

Here, we characterized cultured RPE from AMD donors and controls and showed that AMD RPE exhibit distinct pathological alterations and functional deficits compared with normal RPE.

In concordance with our EM observations showing AP accumulation, Wang *et al.*^44^ reported that drusen in AMD eye sections contained increased levels of autophagic markers. Similarly, increased flavoprotein fluorescence, suggesting elevated mitochondrial dysfunction, has been observed in nonexudative eyes with AMD.^45^ A recent study showed that a marine n-3 polyunsaturated fatty acid that induces a transient increase in cellular ROS levels in RPE activates the oxidative stress response regulator NFE2L2/NRF2 and simultaneously increases intracellular protein aggregates containing p62 and autophagy.^46^

We also observed that the space between the two membranes of the RER to be wider and irregular in AMD RPE (Figure 2b and inset 2d), whereas it appeared rather uniform in normal RPE (Figure 2c and inset 2c). The double membranes of APs are derived from ribosome-free portions of the endoplasmic reticulum (ER)^47^ and a number of pathological conditions can cause ER stress,^48^ which has a significant role in several pathologies including neurodegenration, diabetes and cancer.^49, 50^ The effect of ER stress in AMD has also been discussed;^51^ VEGF secretion is readily induced by such ER stress, and could also be implicated in lysosomal accumulation of lipofuscin within RPE.^51^

Our gene expression analysis revealed increased APOE expression in AMD RPE relative to normal RPE. APOE is synthetized by RPE and is one component of drusen.^52^ It has been shown that human fetal RPE cultured on porous membranes can secrete deposits that are rich in APOE and that exposure to serum could further result in accumulation of drusen components.^53^ This cell culture system mimics drusen formation and could be used for investigating AMD molecular mechanisms. Our observation on APOE gene expression correlates with accumulation of lipid droplets that we observed in AMD RPE (Figure 3a and Figures 2b and d), and is directly related to AMD disease mechanisms. This increased APOE expression in AMD RPE is an interesting observation and could be analyzed further in a larger cohort of AMD and normal RPE.

### Susceptibility to oxidative stress, increased ROS production and dysfunctional mitochondria in AMD

RPE cells are constantly exposed to light-induced oxidative stress,^54^ that over time may damage RPE tight junctions and disrupt the retinal blood barrier.^55^ Oxidative stress has long been hypothesized as a key factor in the development of AMD.^56^ However, the mechanisms underlying the increased susceptibility to oxidative stress in AMD remain unclear. Here, we have established a chronic oxidative stress condition that allowed us to study cell viability of AMD and normal RPE up to 48 h in the presence of a wide range of H_2_O_2_ concentrations. Our data clearly show that AMD RPE exhibit increased susceptibility to chronic oxidative stress. We further demonstrate that AMD RPE produce increased levels of ROS when exposed to oxidative stress as compared with normal RPE.

It is well established that mitochondria are the major source of ROS production and that excess of ROS also induces mitochondrial damage, which can lead to pathologies.^57^ Recent studies show that dynamic increase in extracellular ATP accelerates photoreceptor cell apoptosis and that ATP induces photoreceptor death and retinal remodeling in rats.^58, 59^ Our measurement of mitochondrial activity in AMD and normal RPE confirmed decreased ATP production by mitochondria, increased ATP production by glycolysis in AMD as compared with normal RPE and identified glycolysis as the main source of ATP production in AMD RPE. Consistent with our observations, it has recently been shown that in an early phase of hepatocyte failure, an adaptive metabolic shift, from generating energy predominantly from oxidative phosphorylation to glycolysis occurs. This allows maintenance of energy homeostasis during early stages of liver injury, however, leads to hepatocyte dysfunction during terminal stages of chronic liver disease as hepatocytes are unable to sustain high levels of energy production from glycolysis.^60^

### Dysfunctional autophagy in AMD

Defects in autophagic pathways have been associated with neurodegenerative diseases,^61, 62^ and much interest has focused on the role of autophagy in AMD.^25, 63^

The impact of autophagy on mitochondrial function and accumulation of reactive species has been reported,^24^ and is particularly relevant to degenerative diseases caused by accumulation of oxidative stress over time, where dysfunction in both mitochondrial and autophagic pathways are involved.^24^ APs and mitochondria are suggested to be interrelated homeostatic elements; defects in one element likely impair the other, resulting in increased risk for various human diseases.^64^ How these organelles interact and contribute to the pathophysiology of AMD remains unknown.

Recent evidence links the cellular quality control system, in particular autophagy/mitophagy and mitochondrial dynamics with age-related diseases.^65^ Autophagy loss results in mitochondrial dysfunction and accumulation of organelles, proteins and ROS,^66^ and affects cell viability and induces cell death.^67^

In AMD, RPE dysfunctional autophagy/mitophagy could lead to mitochondrial disintegration by affecting the mitochondrial fission/fusion ratio that is crucial for maintaining functional mitochondria.^68^ Dysfunctional mitochondria in AMD RPE can further translate into an increased oxidative stress response and a shift from oxidative phosphorylation to glycolytic ATP production. Furthermore, dysfunctional mitochondria can affect lipid and glucose metabolism resulting in cytoplasmic lipid and glycogen accumulation, two cellular phenotypes that we observed in AMD RPE. A critical role for autophagy in lipid metabolism is reported, with inhibition of autophagy leading to increased triglyceride storage in lipid droplets.^22^ These observations could also explain the accumulation of lipid droplets in AMD RPE because of dysfunctional autophagy.

Another possible explanation for difference in autophagic flux, stress response and glycolytic ATP production, could be due to an overactive mTOR pathway in AMD RPE. Studies have shown direct control of mitochondrial function by mTOR^69^ with inhibition of mTOR enhancing aerobic glycolysis.^69^ In addition, p62 is suggested to be a key regulator of nutrient sensing in this pathway ^70^ with overexpression favoring more efficient activation of the mTOR pathway.^70^ Our observed increased p62 levels during starvation in AMD RPE could impact mTOR activity. Although the mTOR pathway is important for mitochondrial activity and biogenesis,^71^ overactive mTOR could inhibit autophagy/mitophagy^72^ and subsequently induce accumulation of damaged mitochondria in AMD RPE. Further studies need to be performed to elucidate these interactions in AMD RPE.

Our observation of glycogen accumulation and autophagic dysfunction in AMD RPE is in accordance with recent studies revealing that glycogen accumulation accounts for the neurodegeneration, as well as impaired autophagy.^73^

A recent study reported on increased markers of autophagy and exosomes in the drusen of AMD donor eyes and speculated that increased autophagy and release of intracellular proteins via exosomes by the aged RPE may contribute to drusen formation and AMD.^44^ Consistent with these morphological observations, we found an increased number of APs in AMD compared with normal RPE. However, mechanistic studies analyzing the ratio of LC3-II/LC3-I under starvation revealed that despite AP accumulation, the efficiency of autophagy is reduced in AMD RPE, and that these cells failed to induce autophagy in response to starvation, contrary to what was speculated by Wang *et al.* In addition, analysis of p62 levels further revealed lower autophagic flux in AMD compared with normal RPE. This failure to induce autophagy in response to starvation stress could be due to the difference in basal autophagic flux in AMD as compared with normal RPE. Furthermore, LAMP-1 immunostaining clearly demonstrated swollen vesicles likely indicative of dysfunctional autophagolysosomes in AMD RPE.

Our studies identify specific disease phenotypes in a novel model for human AMD, and suggest that dysfunctional autophagy in RPE contributes to the pathophysiology of AMD.

## Materials and methods

### *In vitro* disease model

A total of 10 eyes from 5 organ donors (2 males and 3 females) clinically diagnosed with AMD and 10 eyes from 5 clinically normal male donors (5 male) were purchased from National Disease Research Interchange^74^(NDRI, Philadelphia, PA, USA). We excluded eyes of donors with other ocular diseases or diabetes from the study. The average time of postmortem enucleation was 9 h and eyes were received in <24 h. Samples with infectious diseases were excluded by serology tests performed by NDRI (Table 1). RPE from macula region were isolated according to established protocols^30^ and cultured in serum-free media^75, 76^ under controlled oxygen (5%) and CO_2_ (5%) conditions.^77^ Studies have shown that low (physiological) oxygen concentration promotes RPE growth^77^ and better protects from ROS-induced damage *in vitro.* RPE were then purified with magnetic-activated cell sorting by positive selection for epithelial cells using anti-BEST1 antibody (Abcam, Cambridge, MA, USA, 1/500) and anti-E-cadherin (Miltenyi Biotech, San Diego, CA, USA, 1/200); and by negative selection using a fibroblast-specific antibody (Miltenyi Biotech, 1/200) to remove fibroblasts. The purity of the sorted cells was confirmed by immunostaining with anti-ZO-1 (1/200) and anti-BEST1, and Real-Time PCR for RPE specific genes (Figures 1a and b). The RPE cells were grown in serum-free RPE media^75^ at 37 ^o^C, with 5% O_2_ and 5% CO_2_.^77^

### SNPs genotyping

RPE were tested for selected SNPs known to be associated with AMD by RT-PCR followed by sequencing analysis.^78, 79^

### Immunostaining

RPE cells grown on transwells 6.5 mm inserts and 0.4 *μ*m membrane (Costar, Corning, NY, USA) were fixed with 4% paraformaldehyde for 10–15 min followed by rinsing with PBS and incubation in blocking/permeabilizing buffer containing 0.5% BSA and 0.2% Tween 20 in PBS. Cells were stained using established protocols for the primary and secondary antibodies. Rabbit and mouse anti-ZO-1 (Invitrogen, Carlsbad, CA, USA) were used at 1/200 dilution. Mouse anti-CRALBP (Novus Biologicals, Littleton, CO, USA), and mouse anti-Bestrophin (Abcam) were used at 1/500. Anti-mouse and rabbit secondary antibodies (Molecular Probes, Carlsbad, CA, USA) were used at 1/1000. Stained cells were mounted with anti-fading medium (Invitrogen), and imaged using Olympus Fluoview microscope (Olympus, Center Valley, PA, USA).

### Quantitative real-time polymerase chain reaction analyses

Total RNA was extracted with the RNeasy kit (Qiagen, Germantown, MD, USA), treated with RNase-free DNase I (Qiagen), and reverse transcribed with oligo-dT using the SuperScript III cDNA synthesis kit (Invitrogen). Quantitative PCR was performed with the QuantiTect SYBR Green PCR Kit (Qiagen). Specific primers (Supplementary Table 1) for each gene were designed with the PrimerQuest software (Integrated DNA Technologies, Coralville, IA, USA), and the cDNA sequences of each gene (GenBank, NCBI, NIH) were used to produce 100–250 bp PCR amplicons that span one or more exon/intron boundaries.

### Electron microscopy

RPE cell cultures were rinsed with Dulbecco PBS (without Ca^2+^ and Mg^2+^), fixed twice in PBS-buffered glutaraldehyde (2.5% at pH 7.4) and PBS-buffered osmium tetroxide (0.5%), and embedded in epoxy resin. Thin sections (90nm) were collected on 200 *μ*m mesh copper grids, dried for 24 h, and double-stained with uranyl acetate and lead citrate. Sections were viewed and photographed with JEOL JM-1010 EM.^80^

### Lipid staining

Accumulation of lipids was assayed with the BODIPY 493/503 lipid-specific dye (Invitrogen) on RPE cultured on 24-well plates or 8-well slide chambers. Stained RPE were imaged using an EVOS FL microscope (Life Technologies, Carlsbad, CA, USA) and the number of green fluorescent lipid aggregates was counted in diseased *versus* normal RPE cells.

### Glycogen concentration assay

Cytoplasmic accumulation of glycogen was assayed using the Glycogen Assay Kit (Sigma, St. Louis, MO, USA) on RPE monolayers grown in 96-well plates. Glycogen concentration was determined by a coupled enzyme assay, which produces a colorimetric (570 nm)/fluorometric (535/587 nm) product, proportional to the glycogen present.

### Oxidative stress conditions

RPE were cultured at 80–90% confluency in 96-well plates, at 37 ^o^C with 5% O_2_ and 5% CO_2_. Oxidative stress was induced with different concentrations of H_2_O_2_ ranging from 0 to 10 mM for 24 or 48 h, followed by cell viability measurements.

### Cell viability assay

Cell viability of RPE monolayers on 96-well plates was measured under different concentrations of H_2_O_2_ (0–10 mM) following 30-min incubation with the PrestoBlue Reagent (Life Technologies); six wells used for each sample. Fluorescence measurements were performed using an Ultra 384 plate reader with 535nm excitation and 612 nm emission wavelengths. The difference between cell viability in normal RPE and AMD RPE was significant between 0.2 to 0.6 mM of H_2_O_2_. At 0.8 mM, most cells died and no significant difference was observed between the diseased and normal RPE. We found that the most significant difference was obtained at 0.4 mM of H_2_O_2_. However, the difference in cell viability was also significant at 0.3, 0.5 and 0.6 mM of H_2_O_2_. Therefore, for the experiments under stress conditions we used 0.4 mM of H_2_O_2_.

### ROS measurement

Production of ROS under oxidative stress was measured using the OxiSelect™ Intracellular ROS Assay Kit (Cell Biolabs, San Diego, CA, USA). RPE monolayers were cultured in 96-well plates in serum-free RPE media^76^ for 20 h and then loaded with 1 mM of the cell-permeable fluorogenic probe 2'-7'-dichlorodihydrofluorescin diacetate for 1 h. Oxidative stress was induced with 2-h incubation in 0.4 mM H_2_O_2_. The fluorescence intensity of each sample, proportional to the ROS levels, was measured against the fluorescence of the provided standard at the indicated time-points using a Tecan (Morrisville, NC, USA) Ultra 384 plate reader.

### Measurement of ATP levels

ATP levels of the RPE monolayers were measured with the Mitochondrial ToxGlo Assay (Promega, Madison, WI, USA). To assay the mitochondrial activity in AMD and normal RPE, the ATP measurement was performed following 2-h incubation with or without 10 *μ*M of the bromopyruvate analog (3-BrPA), an inhibitor of the glycolytic enzyme hexokinase II (EMD Millipore, Billerica, MA, USA).

### Autophagy dynamics assay

For measurement of LC3-II/LC3-I ratios, normal and AMD RPE cells were pre-treated with IGF-1 for 1 h and starved in HBSS for 4 h in the presence or absence of IGF-1 (50 nM). Lysosomal inhibitors E64d and Pepstatin A were added in the last 2 h of starvation at a concentration of 10 *μ*g/ml and the cells were lysed and analyzed with immunoblot for LC3 and p62 based on the established protocol.^35^

### Antibodies

#### (a) Primary antibodies

The primary antibodies used were as follows: rabbit anti-LC3, rabbit anti-p62/SQSTM1 and rabbit anti-beta actin from Cell Signaling Technology (Danvers, MA, USA) used at 1/1000, 1/1000 and 1/10 000, respectively; mouse anti-LAMP-1 from BD Biosciences (San Jose, CA, USA) (1/200); mouse anti-BEST1 from Abcam (1/500); mouse anti-CRALBP from Novus Biologicals (1/500); mouse anti-ZO-1 from Invitrogen (1/200); fibroblast-specific antibody (conjugated to magnetic beads, 1/200), mouse anti-E-cadherin (CD324, 1/200) from Miltenyi Biotech.

#### (b) Secondary antibodies

The secondary antibodies used were as follows: goat anti-rabbit HRP-linked from Cell Signaling Technology; donkey anti-mouse Alexa488-linked and donkey anti-mouse Alexa594-linked from Invitrogen; and anti-mouse IgG MicroBeads from Miltenyi Biotech.

### Immunoblot analysis

Protein samples were extracted in radioimmunoprecipitation assay (RIPA) buffer (1% NP-40, 0.5% sodium deoxycholate, and 1% SDS in 1x PBS), containing freshly added protease and phosphatase inhibitor cocktail tablets (Roche Applied Science, Indianapolis, IN, USA), 1x Protease Inhibitor Cocktail Set I (EMD Millipore), 1 mM sodium vanadate, 50 mM sodium fluoride and 1 mM PMSF (Sigma-Aldrich, St. Louis, MO, USA). Protein concentrations were measured by Bradford assay (Bio-Rad, Hercules, CA, USA). Protein samples were analyzed using the NuPAGE electrophoresis and XCell western blot system (Invitrogen). Primary and secondary antibodies were used based on the manufacturer's instructions. Immunoreactive protein bands were visualized by the Clarity chemiluminescent substrate (Bio-Rad) followed by revealing with X-ray films. Densitometry was performed using the ImageJ software (https://ImageJ.nih.gov).

### Statistical analysis

Quantitative functional and gene expression assays were performed three times, each sample represented by 3–6 replicas per experiment. Mean averages±S.D. were calculated. The statistical significance of the observed differences was tested by ANOVA analysis followed by Tukey's test in the experiments where comparisons were made between groups of samples (i.e., control *versus* AMD) or by *t-*test in the experiments where two samples were compared, using *P*-value<0.05.

## Figures and Tables

**Table 1:** Genotyping and clinical information of AMD and control RPE

**Donor Id#**	**Donor age (gender)**	**Clinical diagnosis**	**CFH (C: risk)**	**HTRA1 (A: risk)**	**LOC (T: risk)**	**Factor B (T: protective)**	**C2 (C: protective)**	**Smoking**	**Cause of death**	**Enucleation (h)**
006	72 (M)	CONTROL	**C**T	**A**G	G**T**	CC	GG	Quit in 1993	Chronic obstructive pulmonary disease	12
010	80 (M)	CONTROL	**CC**	**A**G	G**T**	CC	GG	Quit in 1984	Acute myocardial infraction	9.4
015	11 (M)	CONTROL	TT	**A**G	G**T**	CC	GG	No	Multiple trauma	9.5
023	17 (M)	CONTROL	**C**T	GG	GG	C**T**	GG	N/D	Motor vehicle accident	9
025	50 (M)	CONTROL	TT	GG	GG	CC	GG	No	Myocardial infraction	17
009	68 (F)	AMD	TT	GG	GG	**TT**	GG	2 ppd for 40 years	Stroke	3
014	82 (M)	AMD	**C**T	**A**G	G**T**	CC	GG	1 ppd for 40 years	Cardial related	12
017	81 (M)	AMD	**CC**	**AA**	**TT**	CC	GG	N/D	Cardial arrest	10.5
019	80 (F)	AMD	**C**T	GG	GG	CC	GG	No	GI bleed	9
032	75 (F)	AMD	**C**T	**AA**	**TT**	CC	GG	No	Pancreatic cancer	7

Abbreviations: F, female; M, male

The five clinically diagnosed AMD donors and five clinically normal donors (control) from which primary RPE cultures were established. The cause of death and time of enucleation are indicated. Genotyping data for known AMD-associated Single Nuclear Polymorphisms showing the haplotypes of each donor, carrying risk or protective alleles as marked in bold entries

## Supplementary Material

Supplemenetary Table 1
