# Supplementary material for: Dysfunctional autophagy in RPE, a contributing factor in age-related macular degeneration
Source: Cell Death Dis. 2017 Jan 5;8(1):e2537–. doi: 10.1038/cddis.2016.453 (PMC5386365; doi:10.1038/cddis.2016.453)
Supplement: Supplemenetary Table 1 [file cddis2016453x1.doc]

| **Supplementary Table 1.**  **Primers used in qRT-PCR** |
| --- |

| **Primer Name** | **Primer Sequence (5'-3')** |
| --- | --- |
| POU5F1 FOR  POU5F1 REV | CTGTCTCCGTCACCACTCTG  TGTGTTCCCAATTCCTTCCTTAG |
| NANOG FOR  NANOG REV | CCTGAACCTCAGCTACAAACAGGT  CACACCATTGCTATTCTTCGGCCA |
| LIN28 FOR  LIN28 REV | CGGGCATCTGTAAGTGGTTC  GTAGGTTGGCTTTCCCTGTG |
| MYC FOR  MYC REV | AGCGACTCTGAGGAGGAACAAGAA  CGTAGTTGTGCTGATGTGTGGAGA |
| RPE65 FOR  RPE65 REV | CTGTCCTCGCCGCTCACAGC  GCAGGAGGG CTTGCCCATCAA |
| CRALBP FOR  CRALBP REV | TGTGGGCGGTGAGAAGTTCCTTAT  TCTTCTCTTCCACTTGCAGGCTGT |
| SILV FOR  SILV REV | GCCTGGCAGTGGTCAGCACC  CGGGGTAGACGCAGCCAGTGA |
| PAX6 FOR  PAX6 REV | ACCAATTCCACAACCCACCACA  TGCCCATTGGCTGACTGTTCAT |
| MITF FOR  MITF REV | TTCACGAGCGTCCTGTATGCAGAT  AGTTTCCCGAGACAGGCAACGTAT |
| OTX2 FOR  OTX2 REV | AAGTTCCACTGCTCCAAACCCA  ACTCAGCCCATTGACTGCGTAA |
| RAX FOR  RAX REV | TTTCACCACGTACCAGCTGCAT  TGCAGCTTCATGGAGGACACTT |
| SIX3 FOR  SIX3 REV | TCACTCCCACACAAGTAGGCAA  CGGCCTTGGCTATCATACATCACA |
| VEGFA FOR  VEGFA REV | AAGGAGGAGGGCAGAATCAT  ATCTGCATGGTGATGTTGGA |
| LHX2 FOR  LHX2 REV | TGTTTCAGCAAGGACGGTAGCA  GCACGTGAAGCAGTTGAGGTGATA |
| PEDF FOR  PEDF REV | TGTGCAGGCTTAGAGGGA  GTTCACGGGGACTTTGAA |
| APP FOR  APP REV | TGTCCAAGATGCAGCAGA  CCGATGGGTAGTGAAGCA |
| APOE FOR  APOE REV | GGTCGCTTTTGGGATTACCT  TCCAGTTCCGATTTGTAGGC |
| CRYAA FOR  CRYAA REV | CGGGACAAGTTCGTCATCT  CTTGTGCTTTCCGTGGATC |
| CRYGS FOR  CRYGS REV | TGTGGTCCAAATAGGCAT  CACTGTGGCGAGCACTGT |
| GAPDH FOR  GAPDH REV | GAGTCAACGGATTTGGTCGTAT  AATGAAGGGGTCATTGATGG |

|  |  |  | 1/100 |
| --- | --- | --- | --- |
